# Forgivingness Across Social Relationships and Family Generations Over Time

**DOI:** 10.1111/jopy.70045

**Published:** 2026-01-05

**Authors:** Mathias Allemand, Gabriel Olaru, Patrick L. Hill

**Affiliations:** ^1^ University of Teacher Education Schaffhausen Switzerland; ^2^ University of Zurich Switzerland; ^3^ Charlotte Fresenius Hochschule Germany; ^4^ Washington University in St. Louis Missouri USA

**Keywords:** contextualized forgivingness, family generations, forgivingness, longitudinal data, social relationships

## Abstract

**Introduction:**

This study addressed individual differences in forgivingness, or the tendency to forgive others, across multiple social relationships and family generations over time.

**Methods:**

We used longitudinal data over 4 years across three family generations, including young adults (*n* = 501; *M* = 20.4 years), their parents (*n* = 350; *M* = 50.9 years), and grandparents (*n* = 199; *M* = 76.1 years). Participants were asked to respond on their willingness to forgive the other two generations, as well as their romantic partner and friends. Multilevel modeling was employed to examine how forgivingness differed within persons, family, and generations, as well as over time.

**Results:**

Individual differences in forgivingness were explained by the respondent (62.4%), the social relationship (24.7%), the family (9.6%), and the generation (3.3%). Forgivingness differed depending on the social relationship, with a greater willingness to forgive grandparents and grandchildren. Forgivingness also differed by generation, with the middle and older generations being more forgiving than young adults. Finally, the overall forgivingness increased over time.

**Conclusion:**

The current findings emphasize the importance of contextualizing forgivingness across social relationships and family generations.

## 1 Introduction

Children begin by loving their parents; after a time they judge them; rarely, if ever do they forgive them. – Oscar Wilde

Forgiveness is a virtue widely promoted by most world religions and cultures, and for good psychological reasons. When one forgives, they release themselves from harboring negative feelings and grudges toward the transgressor (e.g., Fernández‐Capo et al. 2017; Worthington 2020). In turn, the act of forgiveness has been linked widely to affective and psychological health benefits (Toussaint et al. 2020; Worthington Jr et al. 2007). Moreover, similar benefits hold for people who forgive by nature. Dispositional forgiveness, known as forgivingness, is associated with greater social, physical, and psychological health (e.g., Hill et al. 2015; Lawler‐Row and Piferi 2006; Rasmussen et al. 2019). However, this literature tends to assess forgivingness with respect to social relationships in general, rather than focusing on specific, personally meaningful ones.

A target‐specific strategy, though, is critical given that it means something qualitatively different to forgive a random stranger, a friend, or even your parent. For instance, as noted by Oscar Wilde, parent‐focused forgivingness may be profoundly different given that parent–child relationships evolve over formative developmental years. Indeed, during the formative years of adolescence, research has shown the potential for willingness to forgive to differ across relationships. In one study, teenage children were shown to differentially forgive mothers versus fathers (Maio et al. 2008). Further support for inter‐relationship differences was found with the adults, as parents were more typically forgiving of their children than of their romantic partners. However, it is unclear whether these differential forgiving tendencies persist into adulthood and how they compare to other relationships outside of the parent–child bond.

The current study employed data from the longitudinal Co‐Development in Personality study (CoDiP; see Bühler et al. 2021; Schaffhuser et al. 2014) to examine the target‐specific nature of forgivingness and its longitudinal stability. This dataset is particularly unique in that it asked three separate generations within the same family about their forgivingness. The current study refers to forgiveness across relationships and generations and defines “generation” based on family roles, encompassing young adults, their parents (middle generation), and their grandparents (older generation)–rather than sociological cohorts or age groups. Distinguishing family generations in this way is essential to avoid conflating generational effects with age or cohort effects. Note that the generational nature of the sample yields differential targets across participants; for instance, grandparents rated their forgivingness of their children and grandchildren while grandchildren would not report on their forgivingness for either of those targets and instead on their parents and grandparents. However, all family generations shared two common targets: friends and romantic partners.

These data allow us to explore three critical questions. First, are adults more dispositionally willing to forgive certain targets more than others? Second, are there age and/or generational differences in forgivingness, when captured in this target‐specific manner? Third, what is the longitudinal stability of target‐specific forgivingness in adulthood? Based on Wilde's conjecture, we would expect relative stability in people's (un)willingness to forgive their parents. However, it is unclear whether stability differs across forgivingness for specific targets. Altogether, addressing these questions will provide substantial guidance on how the field should think about forgivingness as a personality trait dimension.

### 1.1 Forgivingness as a Disposition

Past meta‐analytic work has examined multiple situational and dispositional correlates of forgiveness (e.g., Fehr et al. 2010; Hirst et al. 2019; Van Tongeren et al. 2014). One critical disposition highlighted by this meta‐analysis was forgivingness, showcasing the importance of studying willingness to forgive as a personality disposition (Fehr et al. 2010). Indeed, multiple studies have demonstrated that trait forgivingness predicts a greater propensity for state‐level forgiveness (e.g., Allemand et al. 2007; Stackhouse 2019). This work provides a counterpoint to suggestions that the trait would purely reflect socially desirable responding, particularly when paired with consistent research showcasing the predictive value of forgivingness for personal and relational well‐being (Hill et al. 2015; Rasmussen et al. 2019; Worthington Jr et al. 2007).

Another critical question regarding forgivingness as a disposition has received less attention from the literature. Namely, to what extent does forgivingness exhibit relative stability over time? In line with contemporary views of personality traits (B. W. Roberts 2009), one would not expect perfect stability for forgivingness over time, just as state and trait levels fail to be perfectly correlated. Longitudinal studies of forgivingness are limited in the literature, though extant studies do support expectations of moderate‐to‐high levels of rank‐order stability (Lau et al. 2021, 2022). That said, this work also shows the capacity for individual‐level change in forgivingness over time. This point is further supported by cross‐sectional studies of age trends, which consistently show a modest trend favoring older over younger adults (Allemand 2008; Lawler‐Row and Piferi 2006; Steiner et al. 2011, 2012).

This point may be particularly true with considering the role of forgivingness within family relationships. Familial transgressions are different from events with non‐familial transgressors for multiple reasons. For instance, potential reasons include how these events occur at formative stages of the lifespan, such as adolescence and other periods of earnest identity and social development. Moreover, early transgressions may serve to infringe upon the formulation of trust and security in parent–child relationships. One then may assume that parent‐specific forgivingness may exhibit lower mean levels but higher rank‐order stability relative to other forms of forgivingness. However, again work is limited with respect to the dispositional tendency to forgive specific targets, much less longitudinally (though see Maio et al. 2008; Dewitte et al. 2021).

### 1.2 Target‐Specific Forgivingness

To better understand the value of comparing forgivingness across targets, it is perhaps valuable to consider items in forgivingness assessments. For instance, consider the items presented in Table 1 from the commonly employed Tendency to Forgive Scale (Brown 2003). Typically, such items are assessed without a specific target, allowing researchers to capture forgivingness as a dispositional tendency to forgive and forget, and to avoid harboring grudges when transgressions occur. However, after swapping in specific targets, one can see how respondents may differ substantially in their responses (e.g., “If someone wrongs me, I often think about it a lot afterward” versus “If my parent wrongs me, I often think about it a lot afterward”). People likely develop dispositional tendencies *both* for general forgivingness and forgiving tendencies in certain relationships. However, it remains an open question how closely target‐specific and general tendencies are interrelated.

**TABLE 1:** Forgivingness across generations and targets.

TTF item wording	Young^a^	Middle	Old
1. I tend to get over it quickly when ___ hurt my feelings.	(1) my friends	(1) my friends	(1) my friends
2. If ___ wrong me, I often think about it a lot afterward.	(2) my parents	(2) my children	(2) my grandchildren
3. I have a tendency to harbor grudges when ___ hurt my feelings.	(3) my grandparents	(3) my parents	(3) my children
4. When ___ wrong me, my approach is just to forgive and forget.	(4) my partner^b^	(4) my partner^b^	(4) my partner^b^

*Note:* The items of the Tendency to Forgive Scale (TTF; Brown (2003)) were slightly modified, that is, changing the wording from general (“someone”) to a specific social relationship as the target (e.g., “my friends”).

^a^ The pilot study to test order of presentation focused only on the younger generation.

^b^ The tendency to forgive the partner was only assessed at T2 and T3, and the results of this aspect of forgivingness was reported in Dewitte et al. (2021).

Addressing this question is central to advancing the scientific understanding of the forgiving personality. Forgivingness is commonly viewed as a trait that would influence one's propensity to forgive across situations and transgressors (Brown 2003; R. C. Roberts 1995). However, research has demonstrated the value of moving away from conceptualizing forgivingness as a single, unitary factor. For instance, evidence has accrued that individuals may differ in their willingness to forgive others versus to forgive themselves (e.g., Thompson et al. 2005). Personality research has suggested a related path forward with respect to considering contextualized traits. Past work has demonstrated that contextualized traits, such as conscientiousness *at school* or conscientiousness *at work*, tend to out predict global measures when considering contextualized outcomes, such as student or work success (e.g., Danner and Lechner 2024; Wang et al. 2021; Woo et al. 2015).

A corollary for forgivingness research may be to consider willingness to forgive in the context of certain relationships. People may hold tendencies to forgive that are *dependent on* the circumstances of the transgressor and the situation (Mullet et al. 1998). Specifically, that work found a common factor wherein some individuals were more prone to forgive based on the social consequences and circumstances, including if the transgressor was someone they knew or a family member. When comparing different age groups, the researchers found high levels of endorsement across adulthood for forgivingness of family and people they knew, relative to items reflecting other circumstances. However, this past study only included a couple of items capturing target‐specific willingness to forgive, and it did not consider correlations between these items.

As such, the closest corollary to the current research likely comes from past work with children assessed during adolescence (Maio et al. 2008). In that work, researchers surveyed mother–father‐child triads regarding their willingness to forgive the other two members of the triad. As noted earlier, mean‐level differences were evidenced across relationships, insofar that children were more forgiving of mothers than fathers, and parents were more forgiving of children than their partners. However, two additional aspects of the study are particularly noteworthy. First, participants also completed a measure of general forgivingness, which evidenced, at best, moderate associations with the target‐specific measures. Across all correlational tests (e.g., general‐father, general‐mother, and general‐child for all triad members), associations ranged from *r* = 0.12 to 0.38. Such findings suggest that generalized forgivingness is related, but not perfectly reflective of forgivingness within interfamilial relationships. Second, researchers followed up the family triads 1 year later. Across all target‐specific measures, strong test–retest correlations were evidenced (all *r*s between 0.53 and 0.74). In sum, this study provides initial evidence both that forgivingness may differ across target, and that target‐specific forgivingness exhibits longitudinal stability.

### 1.3 Current Study

The current study added to this research in four critical ways, substantially advancing our understanding of general and target‐specific forgivingness using the CoDiP dataset. First, the sample comprised participants categorized as young, middle‐aged, and older adults. In so doing, the current study provides critical insights into what target‐specific forgivingness looks like in parent–child relationships beyond the potentially turbulent adolescent years. Second, the current study included three generations of family members. As such, the CoDiP dataset provides one of the initial investigations into both forgivingness within parent–child dyads as well as within grandparent–grandchild dyads. The current study also advances beyond the previous work through the ability to compare forgivingness within these familial relationships to participants' willingness to forgive friends and romantic partners. Third, the inclusion of the three family generations of participants allows the opportunity to more comprehensively examine age and generation effects on forgivingness. When evaluating only two generations, any differences between parents' and children's forgivingness could result because parents are older, of a different generation, or because of the different targets of forgivingness. Fourth, the current study investigated the longitudinal stability of forgivingness across three timepoints, spread 2 years apart. Therefore, the current study allows both a more sophisticated assessment of stability, given the additional assessment occasion and the longer timeframe.

## 2 Methods

### 2.1 Transparency and Openness

The current study used data from the longitudinal study Co‐Development in Personality (CoDiP; see Bühler et al. 2021; Schaffhuser et al. 2014, for a detailed description of the study), which was aimed at investigating developmental processes in the immediate family and intimate relationships across three family generations. Approval for the CoDiP study was received from the ethics committee of Basel (approval number: 175/09). No previous study has used these longitudinal data to examine forgivingness across different social relationships and family generations over time. One study examined forgivingness exclusively in relation to the romantic partner focusing on two measurement points (Dewitte et al. 2021). The current research is not preregistered. The analysis code is available on the Open Science Framework (OSF) repository and can be accessed at https://osf.io/5smv3.^1^ All analyses were run in R with the packages *haven* (Wickham et al. 2022), *psych* (Revelle 2022), *lme4* (Bates et al. 2015), and *lmerTest* (Kuznetsova et al. 2017).

### 2.2 Participants and Procedure

Participants were recruited from the German‐speaking region of Switzerland, mainly around Basel and Zurich. In the initial phase, younger, middle‐aged, and older adults were enrolled through presentations of the research project at schools, universities, and universities of the third age—institutions for individuals in post‐retirement life. Recruitment was also supported by advertisements in journals and leaflets. Participants provided contact information for their family members and romantic partners, who were then invited to join the CoDiP study.

To ensure inclusion across family generations, young adults invited their parents and grandparents, while middle‐aged and older adults invited their children and grandchildren. Participants received small incentives such as vouchers, informational brochures with selected results, and seasonal postcards. Students instead earned credits for participation. No monetary compensation was provided. The longitudinal assessments took place at three time points across 4 years: T1 (2010; *n* = 1050), T2 (2012; *n* = 722), and T3 (2014; *n* = 665). At T1, the participants ranged in age from 12 to 92 (*M* = 40.7, SD = 22.4), with 55.8% identifying as female and 44.2% as male. There was a broad range of educational attainment. Of all participants, 20.8% reported having a basic education without an official training qualification, 21.8% had a university entrance qualification, 29.3% had an (advanced) vocational training, and 17.1% completed a university degree or equivalent.

The sample size at T1, along with the age and gender distributions for each family generation, was as follows: young generation (*n* = 501; *M* = 20.4, SD = 3.7; 56.9% women; 1.4% married), middle generation (*n* = 350; *M* = 50.9, SD = 7.1; 56.0% women; 79.4% married), old generation (*n* = 199; *M* = 76.1, SD = 6.7; 60.3% women; 65.3% married). At T1, a total of 1036, 816, 460, 523, and 173 participants reported on the tendency to forgive their friends, parents, grandparents, children, and grandchildren, respectively (partners were not included at T1). At T2, 695, 518, 488, 250, 370, and 117 provided reports with respect to their friends, partner, parents, grandparents, children, and grandchildren, respectively. And finally, 645, 507, 451, 217, 344, and 109 did so at T3. This resulted in an effective sample size of 330.6 (grandchildren) to 1698.6 (friends) when taking the intraclass correlation into account. To test a small effect of β = 0.10 with a significance level of α < 0.05, this resulted in a power of 0.41 (grandchildren), 0.75 (grandparents), 0.76 (partner), 0.82 (children), 0.94 (parents), and 0.99 (friends) (estimated using G*Power; Faul et al. 2007). For a medium effect (β = 0.20), power was high for all targets (≥ 0.94). For the analysis across all targets together, power was very high even for small effects (> 0.99).

### 2.3 Measures

#### 2.3.1 Tendency to forgive

We used the Tendency to Forgive Scale (TTF; Brown 2003) to measure forgivingness across social relationships and family generations. The four items from the TTF were slightly modified to signify the intended target of forgiveness, that is, the partner, friends, and different types of family relationships (i.e., parents, grandparents, children, grandchildren). The items are shown in Table 1. Each of the four items was followed by a 7‐point Likert‐type scale anchored with strongly disagree (1) and strongly agree (7). Several studies provided support for the TTF being a reliable and valid self‐report measure with favorable self‐informant correlations (Brown 2003). In our study, the average Cronbach's alpha reliability estimates for the TTF were at T1: 0.70 (range: 0.53–0.77), T2: 0.72 (range: 0.61–0.82), and T3: 0.73 (range: 0.50–0.83). All relationship‐specific subscales demonstrated acceptable to good Cronbach's alpha reliabilities, except for the “tendency to forgive the grandchildren” subscale, which consistently showed low reliability estimates across all time points (range: 0.50–0.61).

#### 2.3.2 Piloting the measure

We piloted the adaptation of the TTF in a sample of university students and also tested for possible order effects of forgivingness with respect to three different social relationships (targets: friends, parents, grandparents). The sample consisted of 172 students (mean age: *M* = 22.9 years, SD = 3.6, range = 19–40; 84.9% women, 15.1% men). Three groups were each presented a different order of the targets. The reliability estimates and descriptive statistics from this pilot study were as follows: friends (α = 0.79; *M* = 3.74, SD = 1.15), parents (α = 0.72; *M* = 4.03, SD = 1.15), grandparents (α = 0.79; *M* = 4.85, SD = 1.21). In comparison to their friends, participants were more willing to forgive their grandparents (*d* = 0.92) and their parents (*d* = 0.25). Moreover, participants were also more willing to forgive their grandparents as compared to their parents (*d* = 0.63). The order of presentation did not play a significant role (partial η^2^ = 0.006).

### 2.4 Statistical Analyses

To examine the proportion of variance in the tendency to forgive attributable to different sources, we first estimated an unconditional (intercept‐only) multilevel model. The outcome was participants' reported tendency to forgive. Because our design involved repeated assessments across three time points within individuals, individuals nested within families, and responses given for multiple interpersonal targets (e.g., partner, friend, parent, child), we specified a cross‐classified random‐effects structure. Specifically, we included random intercepts for family, individual, generation, and target. The model thus allowed us to decompose the total variance in forgiveness into components attributable to families, individuals, generations, and targets. Variance components were extracted from the fitted model, and intraclass correlations (ICCs) were calculated to quantify the proportion of total variance explained by each level. The equation of the model can be written as:

$$y_{\text{tifr}}=\beta_{0}+u_{i}+v_{f}+\omega_{r}+z_{g}+\varepsilon_{\text{tifr}}$$

with $y_{\text{tifr}}$ representing the tendency to forgive target *r*, at time *t*, by individual *i*, in family *f*. β₀ denotes the overall mean, $u_{i},v_{f},\omega_{r},z_{g}$ random effects of the individual, family, target and generation, and $\varepsilon_{\text{tifr}}$ the residual. In a second step, we estimated the variance decomposition models separately for each target (e.g., partner, friend, parent, child). This allowed us to assess whether the relative contribution of families, individuals, and generations to forgiveness varied depending on the type of interpersonal relationship under consideration. For example, it might be possible that generational do not differ in the tendency to forgive one's friends but do so regarding the partner.

Next, we estimated fixed effects of time, the target, and generation. In contrast to the above, this would provide us with specific effects of the differences across targets or generations (e.g., which generation is most forgiving). The family and respondent were included as random effects on the intercept. As the reference categories across variables, we used the first time point, friends as a common target referent, and the youngest adults. A simplified equation of this model—collapsing the dummy‐coded predictors into single terms—can be written as:

$$y_{\text{tifr}}=\beta_{0}+\beta_{\text{time}}+\beta_{\text{target}}+\beta_{\text{generation}}+u_{i}+v_{f}+\varepsilon_{\text{tifr}}$$

To further investigate potential interaction effects between generation and target, we ran these models separately for each generation and for each target. We opted for this stratified approach instead of including generation × target interactions in the larger model (see equation above) because some targets were completely missing in certain generations (e.g., the older generation did not provide ratings of parents or grandparents). Similar to above, we ran these target and generation‐specific models as it might be possible that generational differences are more pronounced for some targets.

## 3 Results

Descriptive statistics and correlations of the tendency to forgive (TTF) across time and targets (social relationships) are presented in Tables 2 and 3. TTF was similarly stable across targets, with the average 2‐year re‐test being *r* = 0.54, ranging from *r* = 0.47 for grandchildren (T2 to T3) to *r* = 0.64 for the partner (T2 to T3). Furthermore, participants' tendency to forgive was somewhat similar across targets, with an average correlation of *r* = 0.49/0.53/0.51 across all targets at T1/T2/T3. Men were generally more forgiving toward their friends (gender *r* = −0.17), partners (*r* = −0.24), and parents (*r* = −0.21) than women. Similarly, older participants were more forgiving toward their friends (age *r* = 0.15), partners (*r* = 0.16), and parents (*r* = 0.12) than younger participants. On average, respondents participated in 2.14 (SD = 0.88) out of 3 assessments. Women (*r* = 0.17) and older participants (*r* = 0.08) completed more assessments. We did not find any systematic associations between drop‐out rate and the tendency to forgive.

**TABLE 2:** Descriptive statistics for the tendency to forgive across time, generations and gender.

Target	All	Young	Middle	Older	Men	Women
Friends 1	3.93 (1.19)	3.85 (1.14)	3.89 (1.23)	4.20 (1.19)	4.19 (1.16)	3.73 (1.18)
Friends 2	4.05 (1.18)	3.84 (1.13)	4.14 (1.13)	4.41 (1.26)	4.29 (1.17)	3.90 (1.15)
Friends 3	4.18 (1.17)	3.95 (1.08)	4.38 (1.25)	4.35 (1.11)	4.44 (1.19)	4.02 (1.13)
Partner 2	4.02 (1.23)	3.77 (1.18)	4.07 (1.24)	4.35 (1.21)	4.35 (1.14)	3.77 (1.24)
Partner 3	4.27 (1.24)	4.12 (1.18)	4.33 (1.33)	4.46 (1.11)	4.61 (1.26)	4.02 (1.16)
Parents 1	4.11 (1.31)	4.03 (1.29)	4.24 (1.32)	—	4.42 (1.27)	3.88 (1.30)
Parents 2	4.07 (1.23)	3.97 (1.19)	4.26 (1.29)	—	4.45 (1.13)	3.86 (1.24)
Parents 3	4.20 (1.29)	4.10 (1.20)	4.38 (1.42)	—	4.56 (1.26)	4.02 (1.26)
Grandp. 1	4.92 (1.23)	4.92 (1.23)	—	—	5.04 (1.20)	4.82 (1.24)
Grandp. 2	5.04 (1.31)	5.04 (1.31)	—	—	5.33 (1.22)	4.88 (1.34)
Grandp. 3	5.09 (1.20)	5.09 (1.20)	—	—	5.31 (1.08)	4.99 (1.25)
Children 1	4.65 (1.28)	—	4.67 (1.27)	4.61 (1.31)	4.65 (1.28)	4.64 (1.29)
Children 2	4.56 (1.17)	—	4.50 (1.20)	4.67 (1.12)	4.68 (1.12)	4.48 (1.21)
Children 3	4.74 (1.23)	—	4.81 (1.23)	4.59 (1.23)	4.90 (1.17)	4.62 (1.26)
Grandc. 1	5.10 (1.16)	—	—	5.10 (1.16)	5.09 (1.16)	5.10 (1.16)
Grandc. 2	5.18 (1.17)	—	—	5.18 (1.17)	5.35 (1.15)	5.05 (1.18)
Grandc. 3	5.15 (1.16)	—	—	5.15 (1.16)	5.12 (1.11)	5.17 (1.19)

*Note:* Presented are means and standard deviations (in parentheses). Numbers in the labels indicate the measurement occasion. The tendency to forgive one's partner was not measured at the first measurement occasion.

**TABLE 3:** Correlations of the tendency to forgive.

	Gender (f)	Age	FR1	FR2	FR3	PTN2	PTN3	PRN1	PRN2	PRN3	GP1	GP2	GP3	CH1	CH2	CH3	GC1	GC2	GC3
Friends 1	−0.19***	0.10***																	
Friends 2	−0.16***	0.18***	0.51***																
Friends 3	−0.17***	0.17***	0.47***	0.56***															
Partner 2	−0.23***	0.19***	0.46***	0.63***	0.53***														
Partner 3	−0.24***	0.12**	0.39***	0.43***	0.65***	0.64***													
Parents 1	−0.21***	0.09*	0.5***	0.36***	0.36***	0.36***	0.3***												
Parents 2	−0.23***	0.13**	0.34***	0.55***	0.36***	0.6***	0.45***	0.52***											
Parents 3	−0.20***	0.15**	0.41***	0.45***	0.55***	0.45***	0.51***	0.48***	0.54***										
Grandp. 1	−0.09	0.00	0.32***	0.2***	0.18**	0.14	0.16	0.45***	0.32***	0.37***									
Grandp. 2	−0.16**	0.01	0.33***	0.42***	0.26***	0.41***	0.24**	0.35***	0.53***	0.24***	0.48***								
Grandp. 3	−0.12	0.03	0.21**	0.38***	0.37***	0.29**	0.41***	0.37***	0.38***	0.54***	0.41***	0.52***							
Children 1	0.00	0.00	0.52***	0.48***	0.44***	0.40***	0.34***	0.49***	0.34***	0.35***	—	—	—						
Children 2	−0.08	0.07	0.42***	0.61***	0.45***	0.60***	0.42***	0.28***	0.46***	0.31***	—	—	—	0.56***					
Children 3	−0.11*	−0.09	0.39***	0.46***	0.62***	0.48***	0.55***	0.35***	0.33***	0.46***	—	—	—	0.56***	0.59***				
Grandc. 1	0.00	0.11	0.51***	0.34***	0.36***	0.22*	0.44***	—	—	—	—	—	—	0.67***	0.40***	0.37***			
Grandc. 2	−0.13	−0.12	0.4***	0.52***	0.28**	0.40***	0.39**	—	—	—	—	—	—	0.48***	0.64***	0.23*	0.51***		
Grandc. 3	0.02	−0.06	0.28**	0.27**	0.56***	0.24*	0.46***	—	—	—	—	—	—	0.39***	0.37***	0.5***	0.38***	0.47***	
N assessm.	0.17***	0.08**	−0.09**	−0.02	0.00	0.01	0.00	−0.03	−0.01	0.00	−0.03	0.02	−0.02	−0.05	−0.12*	−0.02	−0.14	−0.19*	−0.02

*Note:* Gender (f) = female; N assessm. = number of completed assessments. Numbers in the tables (e.g., Friends 1/FR 1) indicate the time point. The tendency to forgive one's partner was not measured at the first measurement occasion. */**/*** = *p* < 0.05/0.01/0.001. Abbreviations: CH, children; FR, friends; GC, grandchildren; GP, grandparent; PRN, parent; PTN, partner.

**FIGURE 1:**
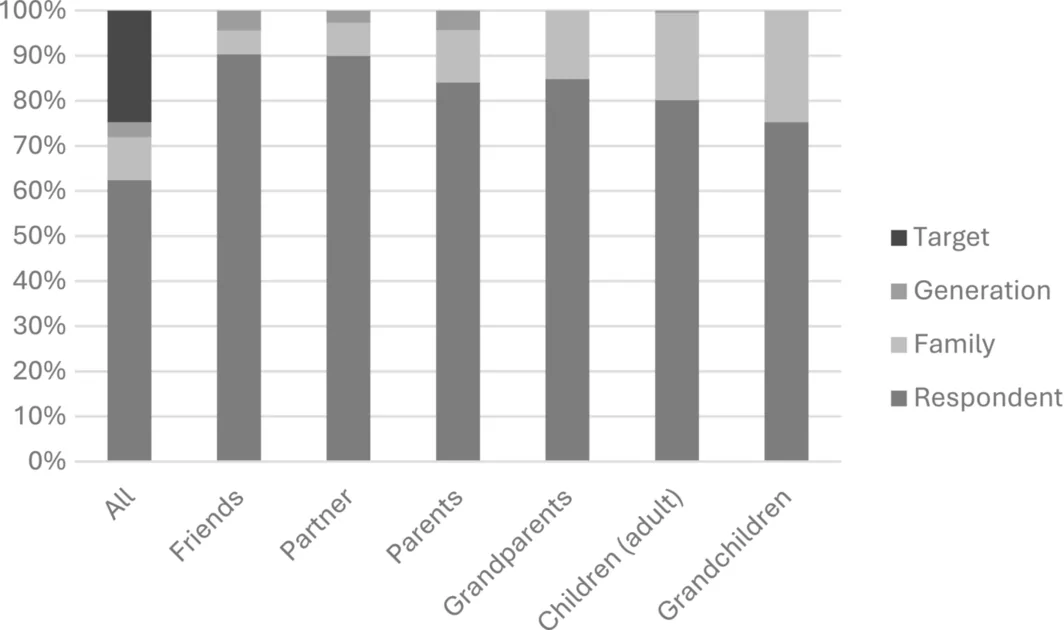
Variance decomposition of the tendency to forgive. Presented is the percentage of the tendency to forgive variance explained by the target (only first column), generation, family, and respondent. This was estimated with an intercept‐only model using the explanation variables as levels. Only the stable variance in the tendency to forgive (i.e., excluding fluctuations across time) is presented here, which amounted to an average of 52.2% of the total variance and was similar across all targets.

The percentage of the stable variance explained by the target, generation, family and respondent is presented in Figure 1. Individual differences in the overall TTF were explained primarily by the respondent (62.4% of variance); 24.7% of variance was explained by the target, 9.6% by the family, and 3.3% by the generation. Looking at the targets separately, the family played the biggest role in the tendency to forgive grandchildren (24.7%), followed by (adult) children (19.3%), grandparents (15.2%), parents (11.7%), the partner (7.3%), and friends (5.3%). The family generation played a comparatively small role for all targets, with percentages of variance explained by generation for each target ranging from 0.5% for children to 2.7% for the partner, to 4.3% and 4.4% for parents and friends, respectively.

Fixed effects of the time, target and generation on the tendency to forgive are presented in Table 4. As can be seen, the TTF increased modestly across time (0.08 SDs per 2 years). The two older generations were both more forgiving than the young adults (middle/older versus younger: 0.24/0.27 SDs). Participants were equally likely to forgive their partner and their friends, but more so their parents (0.14 SDs), children (0.34 SDs), and in particular their grandchildren (0.72 SDs) and grandparents (0.90 SDs). While friends served as the reference category, the non‐overlapping confidence intervals of these estimates suggest that the differences between all targets—except for friends and the partner—are significant.

**TABLE 4:** Multi‐level model estimates for the entire sample.

	*B*	CI	*p*
Intercept^a^	−0.54	[−0.64, −0.44]	< 0.001
Time	0.08	[0.06, 0.10]	< 0.001
Partner	0.03	[−0.03, 0.09]	0.348
Parents	0.14	[0.09, 0.20]	< 0.001
Grandparents	0.90	[0.83, 0.97]	< 0.001
Children	0.34	[0.29, 0.40]	< 0.001
Grandchildren	0.72	[0.62, 0.82]	< 0.001
Middle generation	0.24	[0.12, 0.35]	< 0.001
Older generation	0.27	[0.12, 0.41]	< 0.001

*Note:* CI = 95% confidence intervals; *p* = significance value based on *t*‐tests using Satterthwaite's method. Tendency to forgive scores were *z*‐standardized across the entire sample.

^a^ The intercept represents the tendency to forgive friends in the youngest generation at the first time point.

Results separated by family generations and targets are presented in Tables 5 and 6, respectively. Across generations, we did not find any notable deviations from the previously mentioned general patterns (e.g., all increased across time to a somewhat similar degree). Across all targets, we again found the middle and older generations to be more forgiving than the young adults. The only notable difference across targets was that the tendency to forgive only increased for friends and the partner (0.13 and 0.21 SDs per 2 years, respectively), but not for biological family members.

**TABLE 5:** Multi‐level model estimates for each generation.

	Young			Middle			Old		
	*B*	CI	*p*	*B*	CI	*p*	*B*	CI	*p*
Intercept^a^	−0.50	[−0.62, −0.39]	< 0.001	−0.37	[−0.50, −0.25]	< 0.001	−0.23	[−0.40, −0.07]	0.005
Time	0.06	[0.03, 0.10]	< 0.001	0.11	[0.07, 0.15]	< 0.001	0.07	[0.02, 0.12]	0.004
Partner	0.04	[−0.05, 0.14]	0.345	−0.01	[−0.10, 0.08]	0.828	0.08	[−0.05, 0.21]	0.221
Parents	0.15	[0.08, 0.22]	< 0.001	0.16	[0.08, 0.24]	< 0.001	—	—	—
Grandparents	0.90	[0.83, 0.97]	< 0.001	—	—	—	—	—	—
Children	—	—	—	0.40	[0.32, 0.48]	0.001	0.22	[0.12, 0.33]	< 0.001
Grandchildren	—	—	—	—	—	—	0.68	[0.58, 0.79]	< 0.001

*Note:* CI = 95% confidence intervals; *p* = significance value based on *t*‐tests using Satterthwaite's method. Tendency to forgive scores were *z‐*standardized across the entire sample.

^a^ The intercept represents the tendency to forgive friends at the first time point.

**TABLE 6:** Multi‐level model estimates for each target person.

	Friends			Partner			Parents		
	*B*	CI	*p*	*B*	CI	*p*	*B*	CI	*p*
Intercept^a^	−0.63	[−0.75, −0.52]	< 0.001	0.84	[−1.08, −0.60]	< 0.001	0.32	[−0.46, −0.19]	< 0.001
Time	0.13	[0.09, 0.16]	< 0.001	0.21	[0.13, 0.29]	< 0.001	0.04	[0.00, 0.09]	0.072
Middle generation	0.22	[0.10, 0.35]	0.001	0.18	[0.02, 0.35]	0.033	0.23	[0.09, 0.37]	0.002
Older generation	0.29	[0.13, 0.45]	< 0.001	0.33	[0.11, 0.55]	0.003	—	—	—

	Grandparents			Children			Grandchildren		
	*B*	CI	*p*	*B*	CI	*p*	*B*	CI	*p*
Intercept^a^	0.39	[0.23, 0.56]	< 0.001	0.18	[0.04, 0.33]	0.014	0.47	[0.24, 0.70]	< 0.001
Time	0.06	[0.00, 0.13]	0.059	0.04	[0.00, 0.09]	0.077	0.06	[−0.03, 0.15]	0.214
Middle generation	—	—	—	—	—	—	—	—	—
Older generation	—	—	—	0.11	[−0.29, 0.06]	0.210	—	—	—

*Note:* CI = 95% confidence intervals; *p* = significance value based on *t*‐tests using Satterthwaite's method. Tendency to forgive scores were *z*‐standardized across the entire sample.

^a^ The intercept represents the tendency to forgive the target in the youngest generation that provided ratings (i.e., young adults for all except for children and grandchildren, for which the middle and older generation were used as a reference, respectively) at the first time point.

## 4 Discussion

The current study investigated the tendency to forgive across multiple social relationships and family generations over 4 years. Our findings make four important contributions to the literature on forgivingness. First, in the current intergenerational sample, we found individual differences in forgivingness were explained by 24.7% by the target and different social relationships, respectively, 9.6% by the family, and only 3.3% by the generation. The largest part of variance, with 62.4%, was explained by the respondent, suggesting that the willingness to forgive others depends not only on the targets, but mainly on ourselves. This latter result, in combination with the relatively high average 2‐year re‐test stability of 0.54, demonstrates further evidence for forgivingness as a personality trait. Second, participants differed in their tendency to forgive others depending on their social relationships, with a greater willingness to forgive grandparents and grandchildren. Third, participants also differed in their tendency to forgive others by family generation, with the middle and older generations being more forgiving than young adults. Finally, we found that the overall forgivingness increased over time. To our knowledge, this is the first study to examine differences in forgivingness over time as a function of social relationships and family generations. We will discuss these contributions in detail below.

### 4.1 Social Relationship Differences Matter for Forgivingness

The first research goal refers to the variation of the tendency to forgive across different social relationship differences. It follows work on the assessment of contextualized personality, which often focuses on social roles that reflect characteristic expectations, goals, and behaviors (e.g., Heller et al. 2007; Robinson 2009; Wood and Roberts 2006). Research on contextualized assessments demonstrated considerable cross‐role stability in the personality traits across different social roles such as work, romantic partner, and friend roles, but at the same time also mean‐level differences in traits between social roles. For example, research has shown that across different methodological assessment methods, people are more agreeable as friends than as students or employees (Heller et al. 2007). The current work follows these findings in that 24.7% of individual differences in willingness to forgive were explained by the different social relationships, suggesting that for forgivingness, the social relationship partner does matter.

In addition, the results showed that the tendency to forgive was relatively similar across targets, with a strong average correlation of 0.51 across targets and time points, and that the mean levels of the targets differed. More specifically, the results indicate a nuanced pattern of forgivingness across family relationships. Participants were equally willing to forgive romantic partners and friends, but they showed greater willingness to forgive their parents and children and were even more willing to forgive grandparents and grandchildren. This pattern may reflect varying degrees of emotional closeness and interdependence within families. Prior research suggests that people hold higher expectations for those they are very close to, like romantic partners, because their actions affect shared responsibilities (Harris and Vazire 2016). Romantic partners are usually the main source of attachment and support, with close friends as secondary (Heffernan et al. 2012). As such, any transgressions committed by these individuals may be perceived as more consequential. Our findings support this idea, showing how close relationships influence forgiveness across family generations.

By involving several target persons across three family generations, this work extends earlier work on the topic of forgiveness in family contexts that focused on forgiveness in marriage (Fincham 2019; Fincham et al. 2006; Paleari et al. 2009), and with respect to parents and their adolescent children (Hoyt et al. 2005; Maio et al. 2008; Rivera and Fincham 2015). This latter work on family forgivingness using actor‐perception and partner‐perception models has shown that more than 50% of the individual differences in forgivingness were explained by the respondent, and among children even more than 80% of the variance was explained by the respondent, suggesting that when children are forgiving toward one parent, they are very likely to be forgiving toward the other parent (Hoyt et al. 2005). In our study, 62.2% of the variance in forgivingness was explained by the respondent and 9.6% by the family, suggesting that it also depends on which family you belong to, with some families emphasizing forgiveness more than others or viewing forgiveness as a strength rather than a weakness. It would therefore be valuable for future research to investigate what attitudes and practices lead some families to be more forgiving in general than other families. In addition, shared genes are also a reason for family similarities in forgivingness. For example, a study has shown that the tendency to forgive is moderately heritable (Steger et al. 2007). Since the data also included non‐biological family members such as partners, there is a possibility that people tend to choose partners who are slightly more similar in their personality traits than expected by chance (Lewis and Yoneda 2021; McCrae et al. 2008; Rammstedt and Schupp 2008). However, although family relationships might be critical social contexts for forgiveness, the family context explained relatively little variance as compared to other sources of variance. One reason for this could be that the youngest generation consisted of adult children compared to adolescent children. However, it is also important to note that the families also included partners, so the explained variance could be greater if the family members were purely biological.

### 4.2 Family Generation Differences Matter for Forgivingness

The second research goal refers to the variation of the tendency to forgive across family generations. Although only 3.3% of individual differences in forgivingness tendencies were explained by generation, the results are consistent with the observed positive age correlations with the willingness to forgive friends, partners, and parents in this study, but also with previous work showing positive but modest age correlations (e.g., Fehr et al. 2010). For example, there is evidence for age differences in responses to interpersonal stressors insofar that older adults tend to engage in strategies that minimize negative social experiences and maximize positive ones (Charles and Carstensen 2010; Luong et al. 2011), including the general tendency to react in more forgiving ways with increasing age (Allemand 2008; Steiner et al. 2011, 2012; see Hill et al. 2013 for a review). The age effects in forgivingness might reflect an adaptive response pattern to maintain important social relationships and well‐being and also greater life experience and skills in responding to interpersonal transgressions, and in turn recognition of the importance of forgiving others (Allemand and Olaru 2021).

### 4.3 Forgivingness Changes Over Time

The third research goal refers to the longitudinal study of forgivingness across social relationships and family generations over time. We found an overall increase for forgivingness over 4 years, suggesting that participants became modestly more forgiving over time, and all three generations increased in somewhat similar degree. However, the general increase in the tendency to forgive appears to be driven particularly by increases in the tendency to forgive friends and partners, but not biological family members. Using data from the same CoDiP study and focusing specifically on willingness to forgive the romantic partner between the second and third time points, Dewitte et al. (2021) found a significant, though small, increase in forgivingness across this 2‐year period. The current findings thus significantly contribute to longitudinal research on forgivingness, as less is known about the developmental trajectories of the tendency to forgive in adulthood. One of the few exceptions examined multiple dimensions of forgiveness over 7 years in a large sample of older adults aged 66 years and older (Hayward and Krause 2013). In line with the current findings, the older participants tended to become more willing to forgive. A recent study of Chinese adults examined the longitudinal associations between forgivingness, mood states, and personality traits across three time points over 6 years (Lau et al. 2021). In that study, the authors observed moderate‐to‐high levels of rank‐order stability for forgivingness.

### 4.4 Limitations and Future Directions

Although the current longitudinal study has several strengths, such as a multigenerational sample and the assessment of forgivingness with respect to a broad social context, including romantic partners, friends, and multiple family relationships, the current findings are limited in ways that provide future research directions. First, forgivingness across social relationships was assessed by a self‐report measure. As such, conclusions can only be drawn about people's explicit and self‐perceived willingness to forgive different target persons. Future studies on forgivingness across social relationships and family generations might include target‐perceptions of forgivingness (i.e., how individuals are viewed by the target persons) to complement self‐perception, similar to family forgiveness studies that often use actor‐partner models with self‐ and partner perceptions (Hoyt et al. 2005; Maio et al. 2008). Future research could also benefit from including meta‐perceptions of forgivingness across social relationships and family generations (i.e., how individuals think they are viewed by the target persons), as has been done with respect to personality traits (Schaffhuser et al. 2014)

Second, while the relationship‐specific subscales generally showed acceptable to good reliability, the “tendency to forgive the grandchildren” subscale consistently exhibited low reliability across all measurement occasions. This may be due to the smaller sample size used for this subscale, warranting caution in interpreting these results. Third, relation‐specific forgivingness was assessed without further consideration of particular relationship characteristics, such as satisfaction with specific relationships or frequency of social contact or emotional closeness. Therefore, future research should include relationship‐specific variables and other covariates to help further clarify the present findings. Fourth, due to the cross‐generational design of the current longitudinal study, some targets of forgivingness were generation‐specific (e.g., grandparents as a target for the younger generation, grandchildren as a target for the older generation), which makes comparison difficult. The only targets that were the same across the generations were the willingness to forgive friends and romantic partners. Fifth, although we were able to examine forgivingness over 4 years, any changes between the time points might not be fully captured. Research designs such as measurement burst designs are needed to capture both short‐term manifestations of the tendency to forgive in various social contexts of daily life and longer‐term developmental trajectories of forgivingness over a longer period of time (e.g., Cho et al. 2019). Finally, the current sample was limited to a single country and was largely homogeneous in terms of cultural identity. Therefore, the results may be different in cultures where forgiveness is valued differently (e.g., Sandage et al. 2020). Future research is needed to investigate whether these findings generalize to other contexts, particularly those with lower socioeconomic status and more diverse samples.

## 5 Conclusions

To conclude, the present study advances our understanding of individual differences in forgivingness in several ways. First, the results indicate that characteristics of both the respondent and the target person, as well as the nature of different social relationships, primarily explain individual differences in the tendency to forgive others. In contrast, family and generation appear to play a lesser role. Second, the tendency to forgive differed depending on the social relationships, with a greater willingness to forgive grandparents and grandchildren. Third, the tendency to forgive also differed by family generation, with the middle and older generations being more forgiving than young adults. Finally, the longitudinal results show that the overall tendency to forgive increased over time. Overall, these findings paint a more nuanced and comprehensive picture of forgivingness as a disposition, wherein knowing both who you are *and* who you are forgiving may matter for personality development across adulthood.

## Author Contributions

**Mathias Allemand:** conceptualization, writing – original draft, writing – review and editing. **Gabriel Olaru:** conceptualization, formal analysis, visualization, writing – review and editing. **Patrick L. Hill:** conceptualization, writing – original draft, writing – review and editing.

## Funding

This publication is based on data from the Co‐Development in Personality (CoDiP) Study, funded by the Swiss National Science Foundation (CRSI11_130432).

## Ethics Statement

Approval for the CoDiP study was received from the ethics committee of Basel (approval number: 175/09).

## Conflicts of Interest

The authors declare no conflicts of interest.

## Data Availability

The data that support the findings of this study are available on request from the corresponding author. The data are not publicly available due to privacy or ethical restrictions. See footnote 1 in the paper for details. The analysis code is available on the Open Science Framework (OSF) repository and can be accessed at https://osf.io/5smv3.

## Endnotes

^1^
Because participants participated in families and age could be an identifying variable, there is an increased risk that participants may identify themselves or their family members in the dataset, a problem discussed elsewhere (Finkel et al. 2015). In order to guarantee the anonymity of the participants, we have decided not to publish the available data (see also Bühler et al. 2021).
